# Digital Filtering Techniques for Performance Improvement of Golay Coded TDM-FBG Sensor

**DOI:** 10.3390/s21134299

**Published:** 2021-06-23

**Authors:** Mohamed M. Elgaud, Mohd Saiful Dzulkefly Zan, Abdulfatah A. G. Abushagur, Abdulwahhab E. Hamzah, Mohd Hadri Hafiz Mokhtar, Norhana Arsad, Ahmad Ashrif A. Bakar

**Affiliations:** 1 Department of Electrical, Electronic and Systems Engineering, Faculty of Engineering and Built Environment, Universiti Kebangsaan Malaysia (UKM), Bangi 43600, Selangor, Malaysia; elgaud@ukm.edu.my (M.M.E.); abushagur@ukm.edu.my (A.A.G.A.); p97926@siswa.ukm.edu.my (A.E.H.); hadri@ukm.edu.my (M.H.H.M.); noa@ukm.edu.my (N.A.); ashrif@ukm.edu.my (A.A.A.B.); 2 College of Electrical and Electronic Technology, Benghazi 0021861, Libya; 3 Department of Electrical and Electronic Engineering, Faculty of Engineering, Gharyan University, Gharyan 0021841, Libya

**Keywords:** fiber Bragg gratings, TDM-FBG, Golay codes, moving average, moving median, Savitzky–Golay, signal to noise ratio

## Abstract

For almost a half-decade, the unique autocorrelation properties of Golay complementary pairs (GCP) have added a significant value to the key performance of conventional time-domain multiplexed fiber Bragg grating sensors (TDM-FBGs). However, the employment of the unipolar form of Golay coded TDM-FBG has suffered from several performance flaws, such as limited improvement of the signal-to-noise ratio (SNIR), noisy backgrounds, and distorted signals. Therefore, we propose and experimentally implement several digital filtering techniques to mitigate such limitations. Moving averages (MA), Savitzky–Golay (SG), and moving median (MM) filters were deployed to process the signals from two low reflectance FBG sensors located after around 16 km of fiber. The first part of the experiment discussed the sole deployment of Golay codes from 4 bits to 256 bits in the TDM-FBG sensor. As a result, the total SNIR of around 8.8 dB was experimentally confirmed for the longest 256-bit code. Furthermore, the individual deployment of MA, MM, and SG filters within the mentioned decoded sequences secured a further significant increase in SNIR of around 4, 3.5, and 3 dB, respectively. Thus, the deployment of the filtering technique alone resulted in at least four times faster measurement time (equivalent to 3 dB SNIR). Overall, the experimental analysis confirmed that MM outperformed the other two techniques in better signal shape, fastest signal transition time, comparable SNIR, and capability to maintain high spatial resolution.

## 1. Introduction

For many years, fiber Bragg grating (FBG) sensors have shown their significant sensing abilities. They possess many remarkable advantages over conventional electrical sensors. In addition to their immunity to electromagnetic disturbances, they tolerate harsh environment conditions, embedding ability within the concrete and other composite materials, and multiplexing ability. Over the past few decades, several FBG multiplexing techniques have been proposed to provide quasi-distributed sensing and acquire sensing information along a sensing axis. However, most FBG multiplexing methods have been suffering from many limitations, including expensive and complex components, and limitations in sensing capacity and measurement ranges [1,2].

In this context, the time domain interrogation of low reflectivity FBG sensors (TDM-FBG) can provide the sensing capacity of thousands of sensing points and an extensive measurand range. Furthermore, unlike many other multiplexing techniques, TDM-FBGs are characterized by relatively simple design, cost efficiency, and hardware flexibility [3,4]. Over the past few years, TDM-FBGs have proven their outstanding sensing abilities for various distributed physical quantities, including temperature, strain, pressures, and vibration, at near real-time and off-line performance standards [3,4,5].

Conventional single pulsed TDM-FBGs modulate the intensity of an extremely narrow linewidth laser source to interrogate a cascade of low reflectance FBG sensors. The sensors’ low reflectivity and broad bandwidth allow for low crosstalk levels between adjacent sensors and extendable measurand range. With sufficient pulse energy, the multiplexing capacity of such configurations can reach up to thousands of spatially resolved sensing points along a sensing area of few tens of kilometers [3,5,6]. Furthermore, they can be easily embedded with fully distributed optical fiber sensors (DOFS), such as Raman and Brillouin sensors, for simultaneous static and dynamic measurements [7].

However, many of the TDM-FBG key performance parameters, such as the signal-to-noise ratio (SNR), measurement sensitivity, and range, are associated with the optical power of the probe light. Conventional methods to mitigate these limitations are impractical, and they can be complex, impair spatial resolution, and be time-consuming [3,5,8,9].

Alternatively, it is possible to resolve the trade-off between the optical pulse width, SNR, and spatial resolution by exploiting the significant advantages of pulse compression techniques (PCT) [5,8]. There has been a large amount of literature reporting the utilization of PCT methods based on optical pulse coding scenarios such as Golay complementary pairs (GCP), simplex codes (SC), Walsh codes (WC), hybrid codes (HC), and more [10,11,12,13]. Such techniques resolve the trade-off between the optical power, SNR, and spatial resolution by encoding the probe light with unique coding types and then processing the response signal with a specific pulse compression algorithm. The spatial characteristics of the decoded pulses are identical to those of the conventional single-pulse technique, yet with an enhanced parameter as mentioned above [5,8,10,11]. In this context, the unique autocorrelation properties of GCPs have proven their significant abilities to improve the performance parameters of both semi and fully DOFS. Besides their considerable abilities, multiple formats of unipolar and bipolar GCPs are easy to construct and feasible to be combined with other codes [8,9,11,12]. 

For many years, the deployment of GCPs within the platform of TDM-FBGs has proven its ability to speed up the measurement times, improve the SNR, and extend the measurement range. However, employing the bipolar form of GCPs within the framework of direct detection DOFS such as the standard TDM-FBG sensor is not applicable. Therefore, the bipolar form of GCPs needs to be converted into a unipolar form. However, this procedure is associated with several limitations, including doubling up the number of the deployed codewords, which consecutively slows the processing speed, imposes more noise, and limits the SNIR. Furthermore, the sensing information of the TDM-FBG sensor is encoded within the time domain signals reflected from each FBG. Hence, lacking accurate reads due to noisy background, distortion, broadened signals, or drifted baseline led to insensitive, unrepeatable, and inaccurate measurements [8,9,14,15]. 

Over the past few years, there have been several reports on improving the conventional performance of Golay coded TDM-FBG sensors, including the deployment of interferometric noise suppressing Golay coded optical sources, nesting them with SC technique to improve their shapes and SNIR, and employing their differential form to enhance their spatial distribution and multiplexing capacity [8,9,16]. However, to the authors’ knowledge, proposals for easier-to-implement techniques, such as the digital filtering technique, to further improve the Golay coded TDM-FBG signals have not yet been reported.

Therefore, we propose and discuss the deployment of three types of digital filtering techniques to improve Golay coded TDM-FBG sensor performance. They are moving averages filter (MA), moving median (MM), and Savitzky–Golay (SG). This work is composed of two parts. The first part reports the individual incorporation of Golay codes from 4 to 256 bits with the experimental setup of a standard TDM-FBG of 16 km. The second part investigates the different aspects of enhancement brought to the Golay decoded signals by deploying the mentioned filtering methods. 

## 2. Materials and Methods

### 2.1. Sensing Principle of Single Pulsed and Golay Coded Time Domain Multiplexed FBG (TDM-FBG)

FBG is a type of selective filter made by forming permanent changes to the refractive index of the fiber core. For an incident light of multiple wavelengths, resulted gratings reflect the resonant Bragg wavelength of the grating and transmit the rest. Reflected wavelength must satisfy the Bragg resonant condition and can be expressed as a function of the grating period Λ and the effective refractive index of the fiber core $n_{eff}\;$ [1,2]:

(1) $$\Delta \lambda_{B}=2\;n_{eff}\;\Lambda \;$$

Both the grating period and the refractive index are sensitive to the strain or temperature induced by a local physical source; the shift in the Bragg wavelength can be illustrated as in [1,2]:

(2) $$\Delta \lambda_{B}=\left(\propto +\zeta \right)\Delta T+\left(1-p_{e}\right)\Delta \in$$

where ΔT and Δ∈ are the changes in the temperature and strain, ∝ is the fiber thermal expansion factor and ζ is the thermo-optic coefficient, and $p_{e}$ is the fiber material photoelastic coefficient [1,2,17]. Interrogating the FBG sensors in the time domain is possible by translating the shift in the Bragg wavelength induced by a physical impact into power variation that appears in the peak amplitude of the reflected FBG signals [5]. Apart from the wavelength domain, interrogating the FBG sensors in the time domain reduces the bandwidth requirements of the laser source. Hence, it is possible to deploy a cascade of FBG sensors with the same center wavelength. However, the reflectivity of each should be very low to keep the crosstalk and interference levels between adjacent FBG sensors at their minimum [3,4,5,6].

Furthermore, deploying apodized broad bandwidth FBGs within the time domain platform has provided susceptible performance over extended ranges and quantities [5,7,15,18]. Finally, it is also worth mentioning that the flexibility of the TDM-FBG platform allows for the deployment of several aspects of the FBG sensors other than those listed. For instance, deploying narrow bandwidth FBGs, chirped FBGs, and long-period FBGs (LP-FBGs) is possible by modifying the standard setup of the TDM-FBG and/or incorporating it with other multiplexing and interrogation techniques in hybrid schemes [19,20,21,22,23]. 

**Figure 1:**
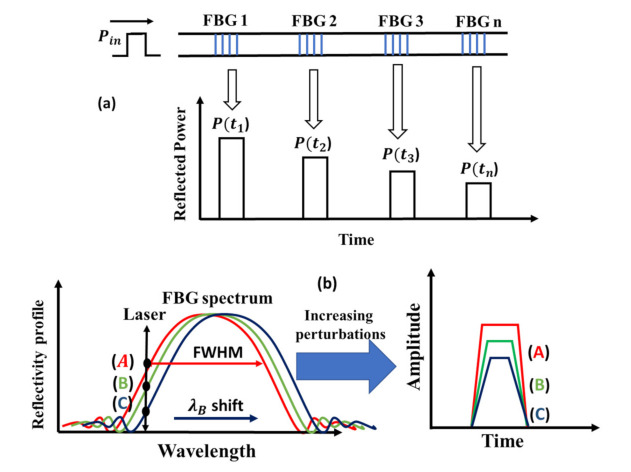
(**a**) Signal representation in TDM-FBGs; (**b**) the translation of Bragg wavelength shift into amplitude variations in TDM-FBG signals.

Figure 1a illustrates the standard schematic of a single pulsed TDM-FBG sensor. The narrow spectrum of a continuous wave (CW) laser is modulated within a short-duration optical pulse and launched into the sensing area. The launched optical pulse with enough energy is capable of interrogating thousands of nearly identical low reflectance FBG sensors [3,5,24]. The FBG reflected signals are then channeled to the receiver section to be detected and distinguished based on their arrival times. In addition, the duration of the interrogation pulse determines the minimum spatial separation between adjacent FBG sensors [3,5].

Figure 1b illustrates the translation of the traditional Bragg wavelength shift induced by physical perturbations from the wavelength domain into amplitude variations in the time-domain FBG signals. Multiple linear shifts in the Bragg wavelength due to physical perturbations are translated into a consistent linear increase or decrease in the received FBG signals, depending on the location of the laser source within the FBG spectrum [5]. In the present example, the laser source’s wavelength is tuned to the left side of the full-wave half maximum (FWHM) point of the FBG spectrum. Positive physical perturbations such as heat impact will induce a red Bragg wavelength shift, i.e., toward the longer wavelength. In this case, the laser source scans the downward region of the FBG spectra; the result in the time domain is a linear decrease in the FBG signal’s amplitudes [5,8,15].

Similarly, negative perturbations induce a blue shift of the Bragg wavelength, meaning that the laser source scans the climb-up region of the FBG spectrum, and the result is a continuous linear increase of the amplitudes of the FBG signals in the time domain [5,15,18]. This principle can be used to detect positive and negative perturbations such as heating and cooling effects in TDM-FBGs. Furthermore, discriminating the cross-sensitivity between dynamic strain and temperature is possible by employing a special assessment of signal processing [5,7].

However, mid and large-scale deployments of conventional single-shot measurement setup suffer from several significant performance limitations, including poor SNR, limited measurand ranges, and low sensitivity [5,15]. Alternatively, it is possible to upgrade this conventional setup to interrogate the sensing area by implementing pulse compression techniques, such as the GCP method, at better performance parameters. Golay coded TDM-FBGs interrogate the sensing area by launching and retrieving multiple traces of coded laser pulses. The unique autocorrelation properties of GCPs provide the decoded signals with significant improvement in SNR and the peak amplitude [8,9,11].

In general, a pair of *A* and *B* codes having an equal length of *L* are said to be complementary if the summation of their autocorrelations provides a perfect correlation function, i.e., one main lobe and zero sidelobes [25], as in [11]:

(3) $$\left(A*A\right)+\left(B*B\right)=2L\delta \left(t\right)$$

The codewords for *A* and *B* can be derived easily by using the recursion method, as in [11,12]:

(4) $$\left(\begin{array}{c} A\\ B \end{array}\right)=\left(\begin{array}{c} A\;|\;B\\ A\;|\;\bar{B} \end{array}\right)$$

For instance, consider a pair of *A* = [1] and *B* = [1]; the next codeword of GCP can be constructed as *A* = [1, 1]; *B* = [ 1, −1] for *L* = 2, and *A* = [1, 1, 1, −1]; *B* = [1, 1, −1, 1] for *L* = 4, and so on [11]. It is worth mentioning that original bipolar GCPs are not suitable for optical systems with direct detection schemes, such as the standard setup of TDM-FBG. Hence, their unipolar format should be introduced before implementing them in such a system [8,11]. This can be achieved by introducing the unipolar format of each bipolar codeword followed by its ones complement. Further explanation of the encoding and decoding of unipolar Golay codes is illustrated in Section 2.3. The SNIR associated with one pair of the bipolar format of *L* bits of Golay codes is equal to $\sqrt{L}$. Since the number of the processed Golay codewords in unipolar setups doubled up, the value of the SNIR is then reduced by half, as in [11,15]:

(5) $$SNIR_{Unipolar}=\frac{\sqrt{L}}{2}$$

### 2.2. Digital Filtering in the Time Domain

The general function of filtering is to remove undesired parts of the signal, such as random noise, or select and extract functional portions of it, lying at a specific frequency range while conserving the crucial portions of the data intact. Digital filters are among the most common signal improvement applications in the time domain. Depending on the application type, digital filters work faster than several conventional algorithms of signal improvement. Digital filters are broadly divided into two main categories, namely finite impulse response (FIR) and infinite impulse response (IIR). However, the filtering process, in general, is the convolution of the time domain signal with the filter function. FIRs are mostly non-recursive filters; they combine delayed portions of the unfiltered signal with feed-forward portions of the un-delayed one. The filter function contains the coefficients of the un-delayed and delayed components of each signal. They work on a small portion of the signal within a finite length, and they do not accumulate errors.

Furthermore, their phase response is linear and easy to implement. However, the implementation of FIR tends to use several coefficients for better performance, which results in a relatively long computational time. The following three subsections brief the working principle of several sliding window FIR filters, namely MA, SG, and MM [26,27,28,29].

#### 2.2.1. Moving Average Filter (MA)

Moving average (MA) filter is an optimal and special case of FIR filter used commonly to regulate an array of sampled data in the time domain. Unlike regular FIR filters, the MA filter utilizes a sequence of scaled ones as coefficients. Thus, the MA filter is significantly helpful for the common signal regulation tasks of reducing the random noise while retaining a sharp step response. MA operates by averaging several samples of the input signal to produce each point in the output signal. The operational equation of the MA filter can be described as follows [28,29]:

(6) $$y\left(i\right)=\frac{1}{r}\overset{r-1}{\underset{j=0}{\sum }}x\left(i+j\right)$$

where x is the noisy input signal, y is the filtered signal and r is the number of samples in the average. In the MA filter, the *i*th value in the data sequence is replaced by the arithmetic mean of all the values in the range *j* = 0 to *j* = (*r* − 1). This window of samples slides forward along the overall range of data points. When *r* is an odd number, the averaging window can be centered precisely at the element *i* in its current position. MA is then symmetrical, and the mean calculations are performed on equally spaced samples within the range *j* = −(*r* − 1)/2 to (*r* − 1)/2 [28,30].

#### 2.2.2. Savitzky–Golay Filter (SG)

MA filters work effectively with local time series with nearly linear changes. However, with data points having extreme changes and more twisted shapes, it is necessary to fit higher-order local polynomials than simple averages. Savitzky–Golay (SG) filtering is a method of data smoothing based on local least-squares and polynomial approximation of the processed data, introduced in 1964 by A. Savitzky and M. Golay. An SG filter reduces the noise and finds a trend line of the noisy input signal by utilizing the least-square fit and a polynomial function as its filter function. SG filtering is a moving window technique as well. The order of the polynomial function is the primary key to the better smoothing features of SG. The larger the size of the window, the better the smoothing performance, as only one set of filter coefficients is required to be calculated. The simplicity of this technique also lies in the fact that the polynomial fitting can be performed by simple convolution with a set of integer weights [29,31,32,33].

Consider that $\left(2M+1\right)$ are consecutive samples on the time series *y* =$y_{1}$, $y_{2}$,$\;y_{3}$…….,$y_{n}$. The polynomial expression $P\left(\tau \right)$ for the input data used by the SG as a filter function can be illustrated as follows [31]:

(7) $$P\left(\tau \right)=\sum_{j=0}^{j=r}\;\beta_{j}\tau^{j}=\;\beta_{0}+\beta_{1}\tau +\ldots \ldots \ldots .+\beta_{r}\tau^{r}$$

where *r* and $\beta_{r}$ are the order and the coefficients of the polynomial function, respectively [31,33]. The coefficient of the polynomial function can be calculated by applying the least-square fit (LSF), which typically minimizes the following expression to obtain the result [31]:

(8) $$\overset{\tau =M}{\underset{\tau =-M}{\sum }}\;{\left[y_{j+\tau }-P\left(\tau \right)\right]}^{2}$$

#### 2.2.3. Moving Median Filter (MM)

The moving median (MM) is a nonlinear FIR filter introduced originally in the 1970s. It is very effective in removing noise while preserving edges of signals and time-domain series. Such filter shows several advantages: edge preservation, robustness against impulsive noise, and efficient noise attenuation. An MM filter moves a sliding window across the data set to be filtered. Commonly, an odd number of samples, S, are sorted within the filtering window, and the element in the middle is used for the filter output. When S is odd, the sliding window is centered about the current position of the element. When S is even, the window is centered at the current and previous elements. Consider a noisy spikey signal $X\left(i\right)$; the MM filtering window S=2K+1, and the filtering output can be illustrated as follows [34]:

(9) $$Y\left(n\right)=MED\left[X\left(n-K\right),\ldots \ldots \ldots \ldots ,\;X\left(n\right),\ldots \ldots \ldots X\left(n+K\right)\right]$$

where $X\left(n\right)$ and $Y\left(n\right)$ are the nth sample of the input and the output, respectively.

Consider that the input signal is of finite length i, consisting of samples from $X\left(n\right)$ to $X\left(i-1\right).$ During the running of the filtering window, some portions of it might fall outside the input signal, i.e., there are not enough samples to fill the window. Therefore, to filter the outmost of the input samples, the window is truncated at the endpoints, and the median is selected over only the samples that already fill the window [29,34].

### 2.3. Experimental Setup

**Figure 2:**
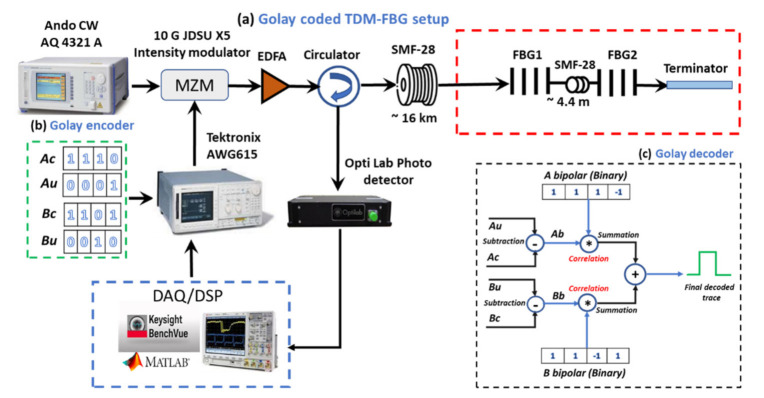
Experimental setup used to perform the 4–256 bit Golay coded TDM-FBG sensor. (**a**) Experimental setup: (**b**) Golay encoder; (**c**) Golay decoder. CW: continuous wave; MZM: Mach–Zehnder modulator; AWG: arbitrary waveform generator; EDFA; erbium-doped fiber amplifier; FBG: fiber Bragg grating; DAQ: data acquisition; DSP: digital signal processing; $A_{u}\;\&\;B_{u}$: unipolar forms of GCP; $A_{c}\;\&\;B_{c}$: their complements.

The experimental setup is schematically depicted in Figure 2a. A continuous-wave (CW) laser source having a linewidth of around 100 kHz, a center wavelength of 1550.5 nm, and an 8 dBm output power was connected to a Mach Zehnder modulator (MZM) for the optical intensity modulation process, with the modulation signal supplied from an arbitrary wave generator (AWG). The AWG generated a series of unipolar Golay codes with a non-return-to-zero (NRZ) format at the sampling rate of 1GS/s, a pulse duration of 10 ns, and a repetition rate of 5 kHz. The intensity-modulated streams of the encoded light were amplified by an erbium-doped fiber amplifier (EDFA: 20dB gain preamplifier) up to around 6 dBm and launched into about 16 km of a standard ITU-T G.652.D single-mode fiber (SMF) optic cable. The end of the fiber spool was spliced with two low-reflectance Gaussian apodized FBG sensors that were spatially separated by around 4.4 m. The two FBGs had nearly the same bandwidth of 2.5 nm, a center wavelength of around 1551.5 nm, and a reflectivity of around 2.5% for FBG1 and 5% for FBG2.

The reflected signals from the sensing area were channeled through the third port of the circulator to a low noise high-speed photodetector (PD). Then, the signal was digitized by the data acquisition system (DAQ) and reserved for further processing and filtering tasks. It should be mentioned that the ambient temperature around the setup was fixed at room temperature during the entire experimental session.

#### 2.3.1. Pre- and Postprocessing of Golay Codes

As mentioned earlier in this paper, each pair of bipolar Golay codes should be preprocessed into the unipolar form. Figure 2b illustrates the unipolar Golay encoder preprocessing of the bipolar form of 4 bits of Golay code. In this section, each bipolar pair was reconstructed into four unipolar codewords by introducing the unipolar form of each pair followed by its ones complement, i.e., {($A_{b}:B_{b}$)} = {($A_{u},A_{c}:B_{u},B_{c}$)} [11]. The four unipolar codewords associated with every codelength of Golay were injected into the sensing area one by one, and their responses were captured and stored for further processing. It is worth mentioning that the optical input power launched into the sensing area was fixed during the entire experiment.

Figure 2c illustrates an example of the decoding process of the 4-bit unipolar Golay code. The stored response of $A_{u}$ was subtracted from that of $A_{c}$ and that of $B_{u}\;$ from $B_{c}$. The results were two codewords in bipolar format. Each of them was then cross correlated with its associated binary bipolar form and summed up to deliver the final response of the measured signal. This process was repeated for the overall set of Golay codes we implemented up to the codelength of 256 bits. To quantify the SNIR, the SNR of all the Golay decoded traces were compared with those obtained from the decoded trace at the codelength of 4 bits.

#### 2.3.2. Processing of Filtering Sessions

Once the signal processing and decoding stages were completed, the decoded signals were imported into the MATLAB platform to perform the digital filtering sessions. The total count of seven decoded measurements associated with the employment of 4 to 256 bits of Golay codes was incorporated individually into the digital filtering setup of MA, SG, and MM, respectively. In this work, we implemented symmetric filtering, i.e., the number of samples, S, for each filter was set to be an odd number of 3, 5, 7, 9, and 11. The multiple filtered traces associated with each session were then processed to analyze their performance parameters.

## 3. Results and Discussion

This section presents the results of the TDM-FBG signals response to the deployment of Golay coding from 4 to 256 bits. This is followed by analysis of the impact of the implemented filtering techniques on the shape, SNR, and the transition duration properties of the decoded signals for all codelengths.

### 3.1. FBG Signal Responses to Increasing Golay Codelength and Multiple Digital Filters

#### 3.1.1. Effect of Golay Codelengths and Digital Filters on the Measured TDM-FBG Signals

**Figure 3:**
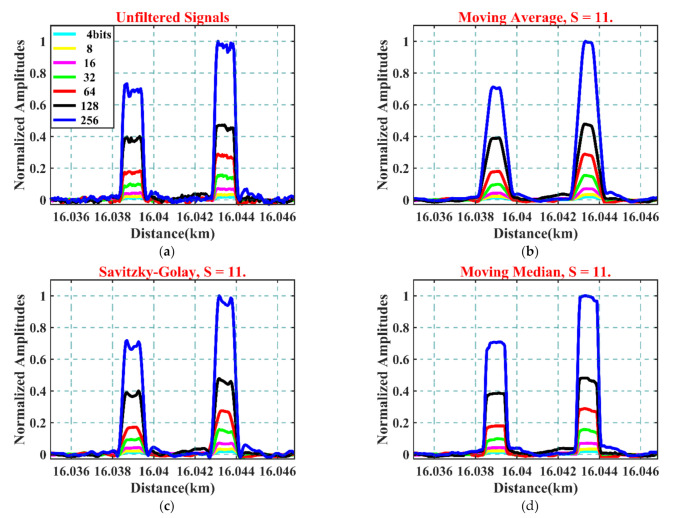
(**a**) FBG signal response to multiple Golay codelengths without filtering; (**b**) with MA, S = 11; (**c**) with SG, S = 11; (**d**) with MM, S = 11.

Figure 3a presents the FBG signal responses to the increasing Golay codelengths from 4 to 256 bits at room temperature with no physical impact applied. The Y-axis illustrates the normalized amplitudes of the decoded signals for every codelength. The X-axis shows the spatial scale translated from the time domain scale, with the exchange factor of about 1 m for 10 ns duration. The signal on the left in every figure is associated with FBG1, which has a lower reflectivity, while the right one is referred to as the second FBG (FBG2), with higher reflectivity. The two signals confirmed the correct encoding and decoding process of the Golay method. The exact location of the two sensors, in addition to the spatial separation of around 4.4 meters, can also be identified. Furthermore, the linear increase of the peak amplitude proportional to each code length was observed. However, the distortion in the peak amplitude for the codelengths of longer than 64 bits was noticeable.

Figure 3b–d present the same decoded signals for all codelengths when S = 11 for the applications of MA, SG, and MM, respectively. From the figures, one can notice that the implemented filters have conserved the key parameters of the standard deployment of TDM-FBG and Golay aspects, such as the spatial properties and the proportional increment of the peak amplitude of the filtered signals corresponding to each length of Golay codes.

The deployment of the MA filter presented in Figure 3b confirmed their excellent smoothing effect. However, compared to the original unfiltered signals case, when S = 11, it also resulted in a noticeable spatial broadening of around 50 cm in the rising and falling edges for both FBGs. The broadening of the pulse width due to the increased sampling size affects the spatial resolution between adjacent FBG sensors; for example, in Figure 3b, the spatial separation between FBG1 and FBG2 was reduced by around 1 m. Furthermore, as expected with such a filter, the overall shape of the filtered signals was converted into a Gaussian-like shape. Further elaborations regarding these points are illustrated in later sections of this paper.

A remarkable smoothing performance with lesser pulse broadening than the MA was recorded when deployed with the SG filter, as illustrated in Figure 3c. Compared to the unfiltered case, SG with S = 11 showed better shape with an acceptable smooth transition toward falling and rising edges. However, the overshoot noises for 128- and 256-bit cases remained.

The smoothing and reshaping performance of the MM filter when S = 11 is depicted in Figure 3d. The FBG signals filtered by this technique showed significant improvement in the shape in terms of sharp transition at the rising and falling edges with excellent forms. Furthermore, one can also observe a substantial decrease in the noise background. Further analysis concerning the impact of the deployed filters on the transition duration properties of the filtered signals is illustrated at the end of this section.

#### 3.1.2. SNR Response to Golay Coding and Digital Filtering Techniques

Further analysis was carried out by analyzing the SNIR values of the unfiltered and filtered signals. Firstly, the contribution of the Golay codes to improve the SNIR was conducted, followed by the analysis of the SNIR of the filtered signals in the case, S = 11. It should be noted that the SNR of each FBG was obtained by dividing the mean value of the FBG signal by the noise amplitude of each trace.

**Figure 4:**
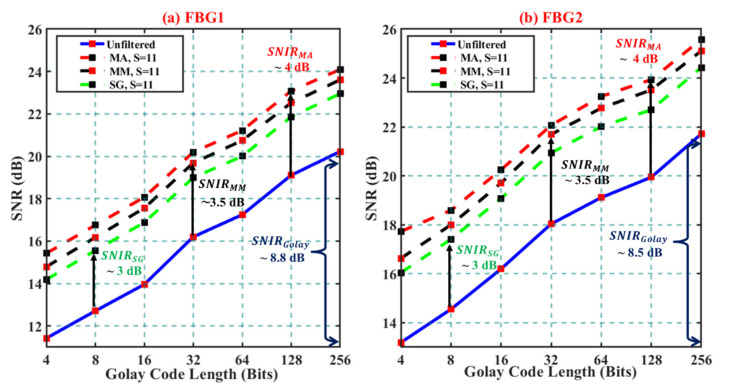
SNR response to increasing Golay codelengths and MA, SG, MM filter when S = 11: (**a**) FBG1; (**b**) FBG 2.

Figure 4a,b illustrate the absolute values of SNR for FBG1 and FBG2 for all filtering techniques, including the SNR of the original unfiltered signals. As expected, the proportional relationship between the SNR and the Golay codelengths was confirmed; 1.5 dB SNR improved for every increased codelength. In good agreement with the theoretical SNIR of 9 dB, compared to the SNR obtained for the 4-bit case, the total SNIR of around 8.8 and 8.5 dB for FBG1 and FBG2 were obtained, respectively.

We then analyzed the SNIR of the signals when filtered with MA, SG, and MM filtering techniques when S = 11. In general, all filtering techniques provided a remarkable SNR improvement, showing a similar linear trend of increasing SNR. Compared to that of unfiltered signals, the incorporation of MA filters improved the SNR of both FBG sensors by around 4 dB. A slightly lesser improvement was recorded through the deployment of the MM filter. Compared to the unfiltered case, an SNIR of around 3.5 dB was recorded for every increase in Golay codelength. The lowest SNR performance was recorded during the deployment of the SG filter; a total SNIR of around 3 dB was obtained. In general, for any codelength, it can be concluded that the incorporation of digital filtering of the Golay coded TDM-FBG signals resulted in at least 3 dB of SNIR, which corresponds to four times faster measurement time compared to that of the unfiltered case.

Furthermore, one can conclude that the deployment of MA, MM, and SG filters into the 256-bit Golay coded TDM-FBG resulted in a further increase in the SNIR values of around 12.5, 12, and 11.5 dB compared to that of the unfiltered 4-bit case.

### 3.2. Effect of Increasing the Number of the Filtering Samples on the Time Properties of 4-Bit Decoded Golay Signal

In general, filtering approaches change many of the critical time domain parameters of the filtered signals, such as their peak amplitudes, time properties, and signal shapes. The degree of these changes is mainly associated with the mathematical properties of the deployed filtering, the number of samples in the filtering window, and the nature of the filtered data. Since many of the vital performance evaluations of the TDM-FBG sensor depend mainly on the time domain properties of the FBG signals, it is mandatory to investigate the impact of the deployed filters on the key parameters of the filtered signals.

In this subsection, we analyzed the impact of the filter type and the increasing number of samples in the filtering window on the time domain properties of the filtered signals. The performance parameters under study are the transition durations and the symmetry of the filtered signals. The former parameter—also known as rising/falling times—was quantified by calculating the transition durations in nanoseconds between the two reference levels, 10% and 90%, of the peak amplitudes of filtered signals [35].

**Figure 5:**
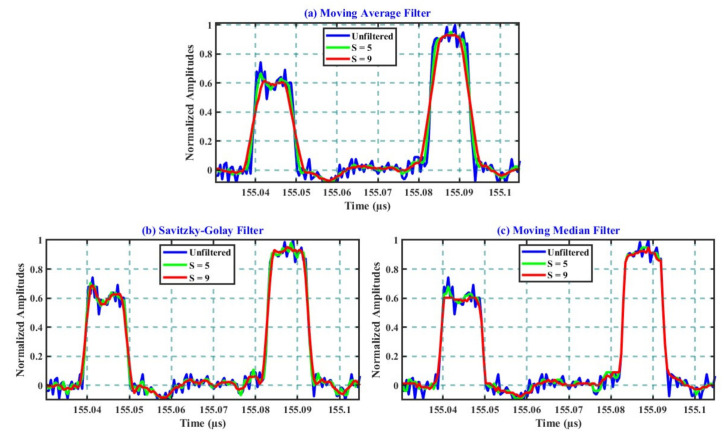
Response of the 4-bit Golay decoded signal to multiple filters: (**a**) with MA, S = 5, and 9; (**b**) with SG, S = 5, and 9; (**c**) with MM, S = 5, and 9.

Figure 5 illustrates the impact of two sampling sizes (S = 5 and 9) on the time properties of the unfiltered 4-bit as representative. Furthermore, Table 1 illustrates the numerical values of the mentioned properties for FBG2 signals analyzed from Figure 5. It should be noted that similar values to the parameters listed for this signal were quantified for FBG1.

Figure 5a–c illustrate the impact of the filtering samples of S = 5 and 9 on the time properties of the filtered signals for the case of the MA, SG, and MM methods, respectively. In each figure, the original unfiltered signal was included for comparison. The unfiltered signal depicted the rising and falling durations of around 1.3 and 1.5 ns, respectively. The unfiltered signal showed a noisy background, few distortions, and slight asymmetry between the two transition durations of around 0.3 ns.

From Figure 5a and Table 1, for the MA case, one can realize the proportional increase in the transition durations and increasing S. For instance, both durations increased nearly equally to around 2.4 ns when S = 5, reaching around 4 ns when S = 9. When S = 11 (as shown previously in Figure 3b), the rising duration of the filtered signal reached around 4.7 ns, while the negative one recorded around 4.5 ns. These long rising and fall durations deteriorate the time properties of the filtered signal, affect its symmetry and shape, and reduce the spatial resolution between adjacent FBGs, consecutively.

Better performance was recorded during the deployment of the SG filter, which is presented in Figure 5b. When S = 5, the filtered signal still conserved the time properties of the original unfiltered signal. However, the transition durations increased up to around 2 ns when S = 9. The most prolonged transitions of around 2.5 ns were recorded when S = 11. In addition to the significant improvement of the signal’s symmetry, one can conclude that the deployment of SG was around 2 ns faster than that of MA. This can be clearly seen by comparing the transition durations of both during the applications of S = 9 and S = 11. In the view of the spatial domain, the 2 ns faster transition duration resulted in shorter spatial (i.e., higher) spatial resolution. Furthermore, the significant ability of the SG filter to preserve and enhance the distortions of the unfiltered signals without significant trade-offs with other key parameters was confirmed.

The impact of the increasing S on the transition properties of the MM filtered signals is illustrated in Figure 5c. When S = 5, as also listed in Table 1, both transition durations increased slightly by around 0.2 ns compared to the unfiltered signal. Similar values of transition durations were obtained during the application of S = 9 and 11. In addition to its remarkable ability to enhance the general shapes of the filtered signals, the analysis confirmed the ability of the MM technique to completely preserve the original transition durations of the unfiltered signals during the entire deployment.

To summarize the results illustrated in this subsection, one can spot the trade-off between the significant smoothing abilities of the MA filter and the time properties of their filtered signals. The proportional increase in the transition durations of their TDM-FBG filtered signals and the increasing S imposed longer spatial separation between adjacent FBGs. Hence, it degraded the multiplexing capacity of the designed sensor. In contrast, the mentioned trade-off was less severe in the deployment of SG. The transition duration of the filtered signals was about 50% faster than that of the MA method. However, when S = 9 and 11, both transition durations of SG were around 1 ns slower than the unfiltered signal. The optimum performance characterized the evaluations of the MM filter. With minimum trade-off, their filtered signals showed the complete conservation of the original signal time properties, together with excellent reshape and smoothing improvements.

## 4. Conclusions

In this paper, we analyzed and compared three digital filtering techniques to improve the performance of the Golay-coded TDM-FBG sensor. The three techniques were moving average (MA), Savitzky–Golay (SG), and moving median (MM). As a baseline for comparison with filtering techniques, the signal-to-noise ratio (SNR) of two unfiltered signals reflected from two FBGs located after 16 km of fiber was measured firstly. By encoding the light with Golay codes from 4 to 256 bits, the SNIR of the FBG signals of around 9 dB was obtained, which agreed well with the theoretical calculations. This also translates into around 60 times faster measurement speeds than of the 4-bit case. Furthermore, this also marks the first experimental deployment of coded TDM-FBG with such a long code length of Golay. Later, the deployment of the filters above was introduced, with the number of samples in the filtering window, S, of 3, 5, 7, 9, and 11. In general, all three filtering techniques improved the sensing performance of the decoded Golay signals compared to the unfiltered ones. In line with the theoretical calculations, all the deployed filters significantly improved the SNIR of the FBG signals. Compared to the case of the unfiltered signals, the highest SNIR of around 4 dB was obtained through the deployment of MA when S = 11, followed by around 3.5 dB for the MM filter at the same value of S. The minimum value of SNIR of around 3 dB was obtained through the deployment of the SG filter when S = 11. Thus, applying the filtering technique alone provided four times faster measurement time for the coded TDM-FBG sensor. Consecutively, incorporating the filtering techniques when S = 11 with 256 bits of Golay codes has shown the minimum SNIR of around 11 dB. This is more than 158 times faster than the unfiltered 4-bit case of the Golay coded TDM-FBG sensor. However, with increasing S, MA delivered the most flawed spatial property. When S = 11, the transition durations on both sides of each of the filtered signals increased by nearly 5 ns. Consecutively, the original spatial separation between adjacent FBGs increased by around 50 cm for both sides of the signals. The deployment of SG showed better spatial performance than the MA one. The maximum increase of both the transition durations was around 2.5 ns (equivalent to 25 cm rising and falling edges in the spatial domain) when S = 11. On the other hand, the deployment of the MM presented the complete conservation of the original spatial properties of the implemented sensor. For all the values of S, filtered signals showed a negligible increase in the transition durations compared to the original ones. Hence, the original spatial separation between adjacent FBGs remained intact. To sum up this comparison, one can mark the MM filter with the optimum overall performance. Filtered signals over this technique have shown the complete conservation of their original spatial properties, improved shapes, and significant SNIRs. The SG filter is marked as the second-best, even though it scored the lowest SNIR values. Both the shape and format of the filtered signals improved significantly compared to the unfiltered ones. Furthermore, the SG signals showed better spatial/time properties than the MA ones. This comparison lists the MA deployment as last. The undesired increase in the time duration properties and general shape of the filtered signals would impair several of the performance parameters of the coded TDM-FBG sensor. Our ongoing work focuses on merging the hybrid deployment of SG and MM filters and machine learning techniques (ML) in several aspects of Golay coded DOFS [36,37].

## Figures and Tables

**Table 1:** Transition duration properties of 4-bit Golay signal associated with FBG2.

S	MA		SG		MM	
	Rising Transition (ns)	Falling Transition (ns)	Rising Transition (ns)	Falling Transition (ns)	Rising Transition (ns)	Falling Transition (ns)
S = 1 (unfiltered)	1.3	1.5	1.3	1.5	1.3	1.5
S = 5	2.4	2.4	1.4	1.5	1.4	1.7
S = 9	4	3.9	2	2	1.4	1.6
